# Supplementary material for: Transcriptional Profile Corroborates that bml Mutant Plays likely Role in Premature Leaf Senescence of Rice (Oryza sativa L.)
Source: Int J Mol Sci. 2019 Apr 5;20(7):1708. doi: 10.3390/ijms20071708 (PMC6480502; doi:10.3390/ijms20071708)
Supplement: Supplementary file 1 [file ijms-20-01708-s001.zip › Supplementary materials/Supplemental Tables 1-3.doc]

**Table S1 List of the primers used in this study for qRT PCR.**

| Gene ID | Gene name | **Forward primer (5´–3´)** | **Reverse primer (5´–3´)** |
| --- | --- | --- | --- |
| *LOC_Os03g50885* | *Actin* | GTGGTCGCCCCTCCTGAAAG | GGCTTAGCATTCTTGGGTCCG |
| *LOC_Os01g12710* | *SAG12* | TGCGGTAAATCAGTTTGCTG | ACGGCGACA TTTTAGTTTGG |
| *LOC_Os02g52650* | *NYC1* | CTGCTTCTCCTGGAATGGTC | CGGATTCTATCAGCCTCTGC |
| *LOC_Os09g26810* | *LHCA5* | GCAACACTGAAGCAACCAAAAT | GTGCCGTCGAGGTGAGGAG |
| *LOC_Os06g44010* | *LHCA2*1* | TGGACGCTGTTCGTGTCGC | CTCCCGTTCTTGATCTCCTTGGT |
| *LOC_Os12g02470* | *ATWRKY40* | CTTCAGGTGCTCGTTCGC | GTTGTGCTCGCCCTCGTA |
| *LOC_Os11g02540* | *ATWRKY53* | TAGCGAGACTGGTAATTGGAGC | GTCTATCACCTGATCGGCACA |
| *LOC_Os07g04560* | *ATWRKY70* | TTCTCCGTCTCCCACTCCG | TGCAGAAGCAGCTTGACATT |
| *LOC_Os10g42130* | *anac042* | GGAGCAATGGAACAGTAGCAG | ATCGTTGCATCCATCCTTGTA |
| *LOC_Os09g36200* | *anac071* | AAAACGAAACCCAAACACT | AGGAGCAATTATGGACGAG |
| *LOC_Os01g21250* | *ATNYE1/SGR* | CGCCATACAGCCTCATCCC | AAAGAATTTAACGCAAAGCAC |
| *LOC_Os01g12710* | *AtLEA5* | AGGGCGGACGAGAAGAAGG | GCAGCCATCCTCTTGGAGTTG |
| *LOC_Os01g28450* | *ATPR1/PR1b* | GTGTGCGGGCACTACACG | CGGCTTATAGTTGCATGTGA |
| *LOC_Os03g08330* | *JAZ10* | CTTCCCCAACTTGCCTCCAA | TGAGTCGTGGGATTTGACGG |
| *LOC_Os01g09800* | *ATNPR1* | CATTCTCTCAAAGGCCGACT | AAGACGTTGAGCAAGTGCAA |
| *LOC_Os06g16370* | *ATCOL2* | TTCTCCTCTCCAAAGATTCC | CATACGCCTTTCTTGTTTCA |
| *LOC_Os01g09450* | *IAA26* | TCGCTCATGCAAGTGGTGAA | CATGTGTGCGCAGCATAGCT |
| *LOC_Os02g57250* | *IAA13* | CAAGGATGGTGACTGGATGCT | GATCCTCAAGCGTTTGCATGA |
| *LOC_Os07g03730* | *PR1a* | CGCCGCAAGTCATGTCCTA | GCTTCGTCTCCGTCGAGTGT |
| *LOC_Os04g32480* | *JAZ5* | CGAGGCAACTAAAGCAAAAGGA | TGAGTGGCTCTTTGGCAAAATT |

**Table S2** Summary of total SNPs in WT and *bml* compared with the reference genome.

|  | WT | *bml* |
| --- | --- | --- |
| Total SNP (No.) | 96228 | 96368 |
| Total INDEL (No.) | 11020 | 10861 |
| SNP_effect_region | | |
| Intergenic region (No.) | 15184 | 15034 |
| 5-UTR (No.) | 13937 | 14149 |
| CDS (No.) | 78446 | 79120 |
| Intronic (No.) | 14247 | 10998 |
| ncRNA (No.) | 5 | 5 |
| 3-UTR (No.) | 33762 | 34457 |
| Splice (No.) | 2132 | 2008 |

**Table S3** The types of nucleotide substitutions in detected homozygote polymorphic SNPs between WT and *bml*.

| Types of nucleotide substitution | WT | *bml* |
| --- | --- | --- |
| Transitions |  |  |
| A->G | 14754 | 14809 |
| C->T | 16242 | 16119 |
| G->A | 15982 | 15946 |
| T->C | 14652 | 14813 |
| Transversions |  |  |
| A->C | 4122 | 4207 |
| A->T | 3564 | 3557 |
| C->A | 5650 | 5320 |
| C->G | 4108 | 4238 |
| G->C | 4107 | 4227 |
| G->T | 5591 | 5279 |
